# Signals of Diagnostic Product Ions of Kavalactones in Their ESI Mass Spectra—Implications for Isomer Differentiation and Identification of Kavalactone Conjugates

**DOI:** 10.3390/ijms27062840

**Published:** 2026-03-20

**Authors:** Małgorzata Kasperkowiak, Wojciech Jankowski, Marcin Hoffmann, Błażej Gierczyk, Rafał Frański

**Affiliations:** 1 Centre for Advanced Technologies, Adam Mickiewicz University, Uniwersytetu Poznańskiego 10, 61-614 Poznań, Poland; malgorzata.kasperkowiak@amu.edu.pl; 2 Faculty of Chemistry, Adam Mickiewicz University, Uniwersytetu Poznańskiego 8, 61-614 Poznań, Poland; w.jankowski@amu.edu.pl (W.J.);

**Keywords:** kavalactones, electrospray ionization, tandem mass spectrometry, fragmentation pathways, isomer differentiation, untargeted analysis

## Abstract

Kavalactones are psychoactive substances that naturally occur in some plants, such as *Piper methysticum*, *Alpinia zerumbet*, and *Achyrocline satureioides*, which are considered to have a significantly positive effect on human organisms. For example, *Alpinia zerumbet* is classified as a life-expanding plant. Although high-pressure liquid chromatography–mass spectrometry has been used for kavalactone analysis in plant material, the fragmentation pathways of protonated kavalactone molecules are not fully known and require further detailed study. In this paper, the fragmentation pathways of [M+H]^+^ ions of twelve kavalactones, including three pairs of isomers, are discussed in detail. Special emphasis has been placed on diagnostic product ions, which are characteristic of kavalactone structures. It has been demonstrated that diagnostic ions and structure–fragmentation relationships enable the differentiation of isomeric kavalactones and may be useful for the identification of other kavalactone conjugates, such as kavalactone dimers or kavalactone glycosides.

## 1. Introduction

Kavalactones are psychoactive substances that naturally occur in some plants; however, in contrast to most psychopharmaceuticals, their influence on living organisms is not considered to be negative. First of all, there are no issues with their withdrawal or addictive effects, and they are free of drug interaction concerns, at least in typical concentrations that can be achieved in humans [1,2,3]. Kavalactones have various positive effects, such as anti-anxiety and cancer-preventing effects [4] and anti-inflammatory properties [5], they can be used as motivation-supporting substances for the intensive training of special forces [6], and they may also increase the female sexual drive [1]. A diet rich in *Alpinia zerumbet*, a plant containing large amounts of kavalactones, can significantly extend the lifespan [7]. Extracts from *Piper methysticum* (commonly known as kava, the plant containing the highest amount of kavalactones), have found many uses for medicinal purposes, during religious ceremonies and social gatherings, in traditional societies in the South Pacific Islands [8,9]. It should be noted, however, that according to the well-known maxim “all things are poison, and nothing is without poison”, the consumption of excessive amounts of kavalactones can be harmful [1,7].

Chromatographic techniques, including both gas and liquid chromatography, with various detection methods, have been successfully used in the analysis of kavalactones [10,11,12,13,14,15,16]. Nowadays, the most popular technique for kavalactones analysis has become high-pressure liquid chromatography–mass spectrometry (HPLC-MS) because of its many beneficial features, such as high sensitivity and specificity [17,18,19], and most importantly, the fact that it enables the analysis of kavalactones together with other compounds, such as flavonoids [20,21,22]. Since most kavalactones do not have acidic groups that could be deprotonated, they have been analyzed in the positive-ion mode as [M+H]^+^ ions. In order to perform the untargeted analysis of kavalactones, it is important to know the fragmentation pathways of kavalactones [M+H]^+^ ions. There are a few papers in which these pathways have been described; however, the presented data are often disputable, to say the least, or incomplete. Warburton and Bristow have studied the fragmentation pathways of six kavalactones using sustained off-resonance irradiation collision-induced dissociation and infrared multiphoton dissociation (SORI-CID and IRMPD) with Fourier transform ion cyclotron resonance mass spectrometry (FT-ICR-MS) [23]. The elemental composition of product ions has been unambiguously determined; however, only the structures of yangonin product ions have been suggested [23]. The product ion assignments have been performed in the work by Boeldijk et al.; however, there are numerous incorrect assignments in their paper (e.g., for dihydrokavain ([M+H]^+^ at *m*/*z* 233), the product ion at *m*/*z* 187 has been attributed to the loss of a ketene molecule) and the product ion structures have not been proposed [24]. The fragmentation pattern of kavain was claimed to be presented in the paper by Jaiswal et al.; however, the proposed product ion structures did not contain the charges and did not match the claimed elemental compositions (both are unacceptable) [25]. Fragmentation patterns of nine kavalactones (among them three flavokavains) were proposed in the paper by Tang and Fields, and this paper seems to contribute the most significantly to the study of the fragmentation of kavalactone [M+H]^+^ ions [26]. Its minor shortcoming is that the high-resolution MS data are reported to two decimal places, while four decimal places are recommended [27], in addition to a few typos (e.g., the exact mass of [C_15_H_19_O_4_]^+^ is 263.1278, so the reported accurate mass, 263.11, does not match the exact mass; additionally, the formula of the flavokavain B [M+H]^+^ ion should be C_17_H_17_O_4_, not C_17_H_16_O_4_, etc.) [26].

**Figure 1:**
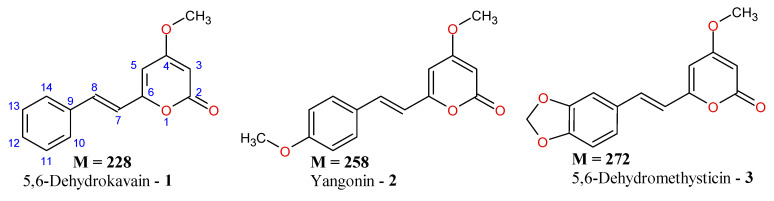

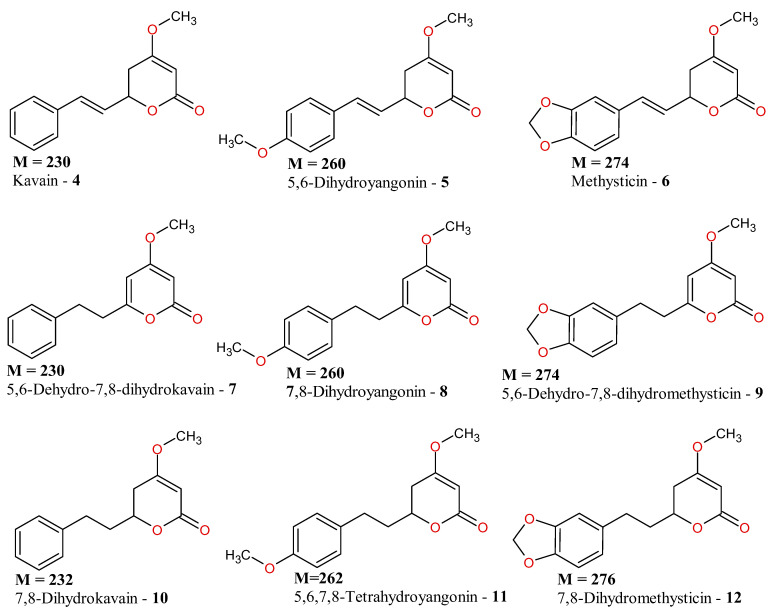
Structures of the studied kavalactones **1**–**12**.

Kavalactones belong to the polyketide class of secondary metabolites and share a common 6-(2-arylethenyl)-4-hydroxy-2-pyrone skeleton, whose most common modifications involve methylation of the C4-OH group; hydrogenation of the C5–C6 double bond and/or the C7–C8 bond; and the substitution of the phenyl ring with hydroxy, methoxy, or methylenedioxy groups. Since the issue of the fragmentation pathways of kavalactone [M+H]^+^ ions required further studies, in this paper, we present a comprehensive study of the fragmentation pathways of twelve kavalactone [M+H]^+^ ions (**1**–**12**), among them three pairs of isomers (Figure 1). The found diagnostic ions and structure–fragmentation relationships enable isomer differentiation and may be useful for the identification of other kavalactone conjugates during untargeted analysis, as also presented in this paper.

## 2. Results and Discussion

### 2.1. Identification of Compounds ***1***–***3***

**Figure 2:**
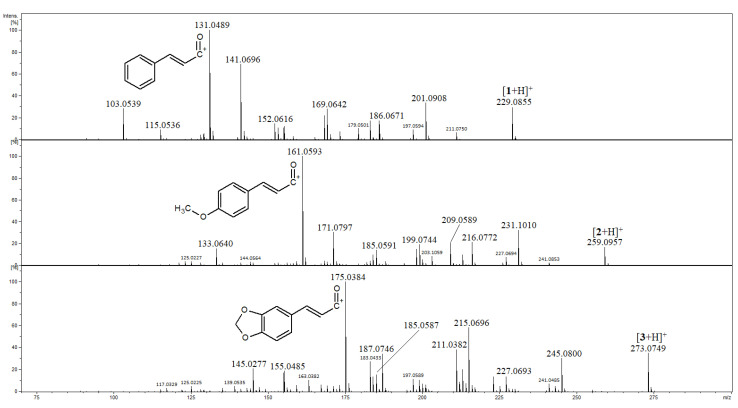
Product ion spectra of compounds **1**–**3**.

**Figure 3:**
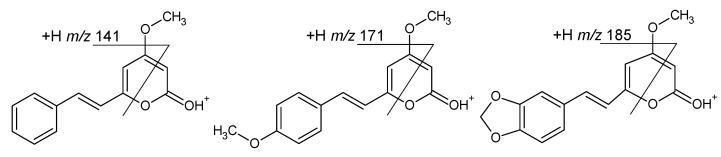
Simplistic mechanism of formation of product ions at *m*/*z* 141, 171, and 185 from protonated **1**, **2** and **3**, respectively.

Figure 2 shows the MS/MS spectra of protonated compounds **1**–**3**. As these compounds have a conjugated system of double bonds, very little fragmentation was observed at a low collision energy (15 eV). At a higher collision energy (25 eV), the most abundant product ions were those at *m*/*z* 131.0489, 161.0593 and 175.0348 for **1**, **2** and **3**, respectively. These product ions can be regarded as diagnostic, indicating the presence of the double bonds C5=C6 and C7=C8. Subsequent decomposition of these ions, consisting in the loss of CO molecule, yielded product ions at *m*/*z* 103 and 133 for **1** and **2**, respectively. For **3**, the loss of CO from the ion at *m*/*z* 175 is a minor process (the product ion at *m*/*z* 147 has very low abundance). The dominant decomposition of the ion at *m*/*z* 175 is the loss of the H_2_CO molecule (in the formation of the product ion at *m*/*z* 145) due to the presence of a methylenedioxy moiety (Figure 2) [28,29]. In the mass spectra shown in Figure 2, there were also peaks of product ions at accurate *m*/*z* 141.0696, 171.0797 and 185.0587 for **1**, **2** and **3**, respectively. Their elemental compositions were deduced as C_11_H_9_, C_12_H_11_O and C_12_H_9_O_2_ (calculated exact *m*/*z* 141.0699, 171.0805 and 185.0597, respectively), and Figure 3 shows a simplistic mechanism of their formation from protonated **1**, **2** and **3**. It is difficult to propose a detailed mechanism for the formation of product ions at *m*/*z* 141, 171, 185, as well as their structures, since there are many possible ones (e.g., their structures may correspond to the [M-H]^+^ ions of benzocycloheptatriene [30]). On the other hand, the formation of these product ions can be regarded as a common feature of fragmentation in [**1**+H]^+^, [**2**+H]^+^, [**3**+H]^+^; therefore, these product ions can be classified as diagnostic ones for compounds which are conjugates of **1**. Tang and Fields have reported the MS/MS spectra of **1** and **2**; however, their spectra were obtained at low collision energies and the product ions at *m*/*z* 131 and 161 were low-abundant, while those at *m*/*z* 141 and 171 were not detected [26].

In the extract of *Piper methysticum*, the *cis* isomer of yangonin (**2**) was also detected (Figure S1). As mentioned in many papers, yangonin is prone to isomerize to its *cis* isomer, and the formed isomer has a shorter retention time [10,24,26], as was also observed in our study (Figure S1). However, the product ion spectrum of *cis*-2 was very close to that of 2 (Figure S3 and Figure 2) [28,29]. In the mass spectra shown in Figure 2); therefore, the differentiation of these isomers by MS/MS experiments seems to be hardly possible.

### 2.2. Identification of Compounds ***4***–***9***

**Figure 4:**
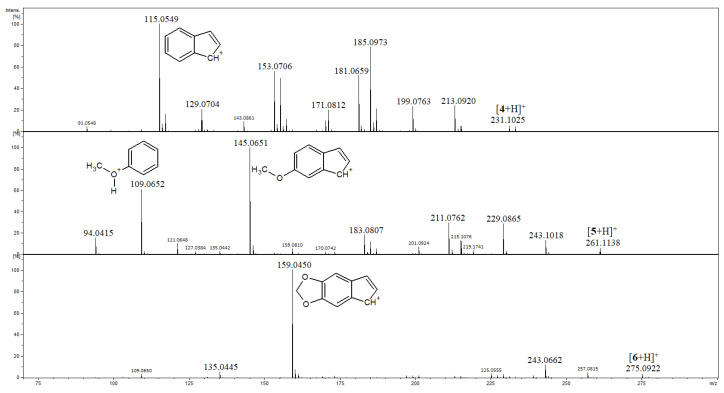
Product ion spectra of compounds **4**–**6**.

**Figure 5:**
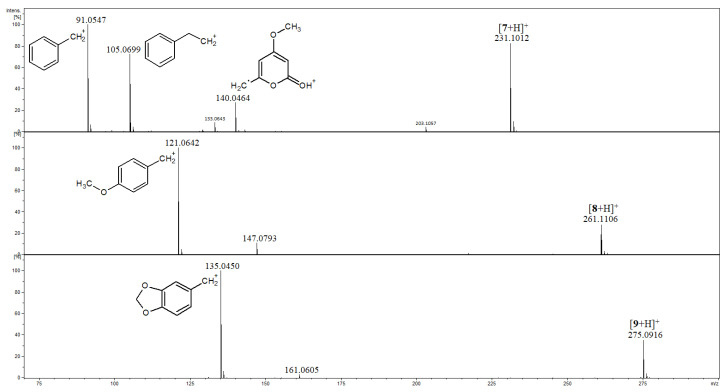
Product ion spectra of compounds **7**–**9**.

Compounds **4**–**9** are three pairs of isomeric kavalactones, namely **4** and **7**, **5** and **8**, and **6** and **9**, and the MS/MS spectra of their [M+H]^+^ ions are shown in Figure 4 and Figure 5. A common feature of fragmentation of protonated **4**, **5** and **6**, which have single C5–C6 bonds and double C7=C8 bonds, is the formation of product ions at accurate *m*/*z* 115.0549, 145.0651 and 159.0450, respectively (Figure 4). Their elemental compositions were deduced as C_9_H_7_, C_10_H_9_O, C_10_H_7_O_2_ (calculated exact *m*/*z* 115.0542, 145.0648, 159.0441, respectively). In the mass spectra of protonated **4**, **5** and **6**, the plausible structures of these ions, which correspond to the [M-H]^+^ ion of indene [31], are presented, and they may be regarded as diagnostic ones for kavalactones containing single C5–C6 bonds. A common feature of the fragmentation of protonated **7**, **8** and **9**, which have double C5=C6 bonds and single C7–C8 bonds, is the formation of benzylium product ions strictly corresponding to *m*/*z* 91.0548, 121.0643 and 135.0450, which are the most abundant product ions, as shown in Figure 5 (calculated exact *m*/*z* 91.0542, 121.0648 and 135.0441, respectively). The ions of this kind, well known in electron ionization mass spectrometry, may isomerize to the seven-membered tropylium ions [32] and they may be regarded as diagnostic ones for kavalactones containing single C7–C8 bonds. Product ions at *m*/*z* 91, 121 and 135 were also detected for compounds **4**, **5** and **6**, respectively, but with very low abundances (Figure 4).

It is worth adding that the MS/MS spectra of **4** (kavain) and **6** (methysticin) reported by Tang and Fields resemble those in Figure 4 very well [26]. However, Tang and Fields did not compare the spectra of **4** and **6** with those of their respective isomers **7** and **9**.

### 2.3. Identification of Compounds ***10***–***12***

**Figure 6:**
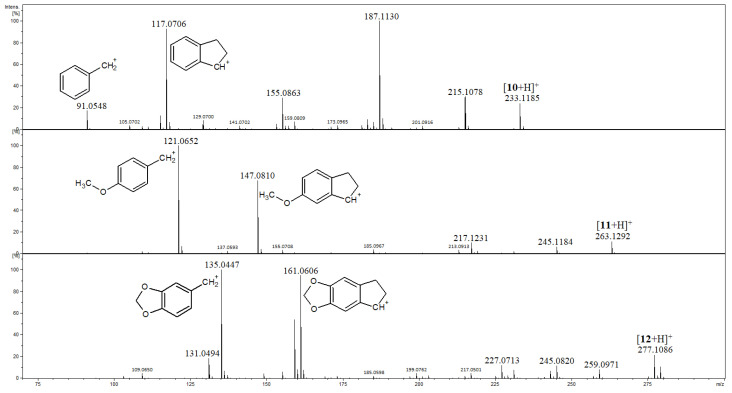
Product ion spectra of compounds **10**–**12**.

Compounds **10**–**12** have the single bonds C5–C6 and C7–C8; therefore, the MS/MS spectra of their [M+H]^+^ ions, as shown in Figure 6, reveal the features of the above discussed compounds **4**–**9** related to the presence of these single bonds. Namely, the peaks corresponding to benzylium product ions at accurate *m*/*z* 91.0548, 121.0652 and 135.0447, which were formed due to the presence of the single bond C7–C8, and those of the product ions at accurate *m*/*z* 117.0706, 147.0810 and 161.0606 (with the calculated exact *m*/z 117.0699, 147.0805 and 161.0597, respectively) most probably correspond to the [M-H]^+^ ion of indane (2,3-dihydroindene) as shown in Figure 6 [33]. The spectra of **10** and **12** are in agreement with those reported by Tang and Fields [26], although in their study, the compound **11** was not included and the structures of the product ions were not proposed.

### 2.4. Other Fragmentation Pathways of Protonated ***1***–***12***

Besides the above described diagnostic product ions, which enabled the identification of compounds **1**–**12**, the MS/MS spectra of these compounds show the peaks corresponding to other abundant product ions, specific to a given compound.

In the MS/MS spectra of **1** and **2**, there are intense peaks of product ions formed as a result of the losses of CO, HCOOH, CH_3_OH, H_2_O molecules and CH_3_^•^ radical, whereas the spectrum of **3** presents intense peaks of product ions formed as a results of the losses of CO, H_2_CO, CH_3_OH molecules, in different orders, since the presence of a methylenedioxy moiety in **3** significantly affects the fragmentation pathways of [**3**+H]^+^ (Figure 2 and Supplementary Material Scheme S1). The loss of CO from [M+H]^+^ ions of **1**, **2** and **3** may be regarded as a characteristic feature of these compounds.

The MS/MS spectra of **4** and **5** reveal the intense peaks of product ions formed as a result of the losses of HCOOH, CH_3_OH and H_2_O molecules (Figure 4 and Supplementary Material Scheme S2), whereas in the MS/MS spectrum of **6**, the corresponding peaks have minor intensities (Figure 2). In the MS/MS spectrum of **5**, there is an abundant peak of an ion that can be described as protonated methoxybenzene (*m*/z 109, Figure 4).

The second most abundant product ion detected for **7** is that at *m*/*z* 105, whereas the corresponding ions were not detected for **8** and **9** (Figure 5). In the MS/MS spectrum of **7**, there is also an intense peak of an odd-electron ion at *m*/*z* 140 formed as a result of the loss of a benzyl radical from the [**7**+H]^+^ ion, and the corresponding ions were also not detected for **8** and **9** (Figure 5).

The diagnostic product ion at *m*/*z* 91 occurs at a moderate abundance for **10**, and the most abundant product ion was formed as a result of the HCOOH loss from [**10**+H]^+^ (followed by the loss of the CH_3_OH molecule). The corresponding ions occur in minor abundances for **11** and **12** (Figure 6, Supplementary Material Scheme S3).

### 2.5. Identification of Kavalactone Conjugates Using Cone Voltage-Induced Fragmentation

The above described fragmentation pathways were checked as to whether they can be successfully used for the identification of kavalactone conjugates under different experimental conditions, namely by using HPLC-MS analysis with cone voltage-induced fragmentation in-source (collision-induced dissociation in-source, CID in-source). Despite the limitations of this technique, which are well known, this technique enabled detailed structure elucidation [34].

**Figure 7:**
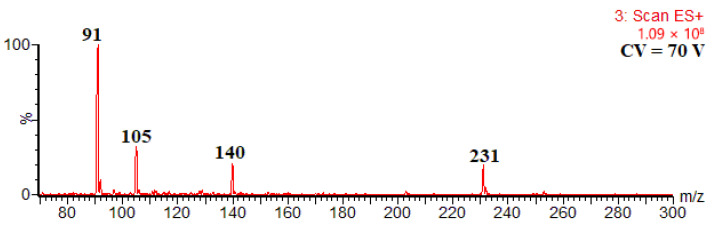
CID in-source mass spectrum of compound **7**.

In the extract of *Alpinia zerumbet*, the main kavalactone found was compound **7** (kavain isomer). Figure 7 shows its ESI mass spectrum obtained upon HPLC-MS analysis with CID in-source. The mass spectrum shown in Figure 7 strongly resembles that obtained for the HPLC-MS/MS condition, including the formation of the unexpected odd-electron ion at *m*/*z* 140 (Figure 5). In other words, the formation of diagnostic product ions from protonated kavalactone molecules is independent of the instrumental conditions.

#### 2.5.1. HPLC-MS Analysis of Kavalactone Dimers

Usually, during the analysis of kavalactones present in the *Alpinia zerumbet*, kavalactone dimers were also detected [5,35,36]. In our extract, we detected three dimers of **1** (or *cis*-**1** isomer) at retention times of 15.5, 15.8 and 16.2 min (**1a**, **1b**, **1c**), as shown in the Supplementary Material (Figures S4–S8).

**Figure 8:**
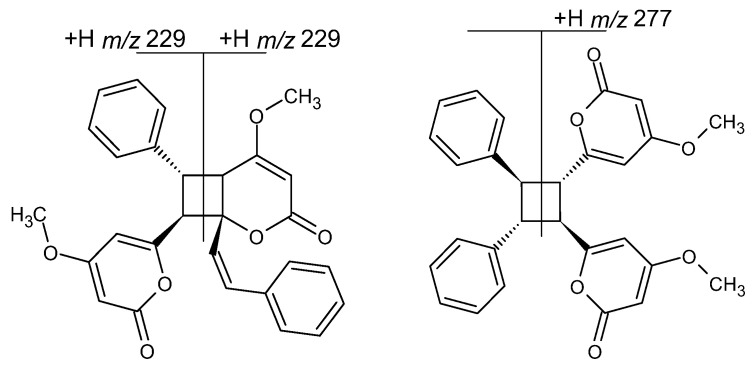
Plausible structures of **1a** and **1c** and their main fragmentation pathways.

The plausible structures of **1a** and **1c** are shown in Figure 8 (obviously, other geometric isomers cannot be excluded). For compound **1a**, an abundant product ion at *m*/*z* 229, formed without the use of CID, has been already observed [36], which is analogous to our results (Figure S6). Its ESI mass spectrum obtained at a high cone voltage (Figure S7) revealed peaks characteristic of **1**, which were formed as a result of the subsequent decomposition of the ion at *m*/*z* 229 (Figure 1). In other words, the fragmentation pathways found for **1** also occur in its dimer **1a**. For compound **1c**, the product ions at *m*/*z* 277 (formally the loss of a stilbene molecule) and *m*/*z* 179 (formally a [stilbene-H]^+^ ion) were detected at a high cone voltage (Figure S8), analogously to the results described elsewhere [36]. For dimer **1c**, the product ions characteristic of **1** were not detected (or occur in very low abundances), which indicates that the formation of product ions found at *m*/*z* 277 and 179 is energetically favored over the other possible fragmentation pathways.

**Figure 9:**
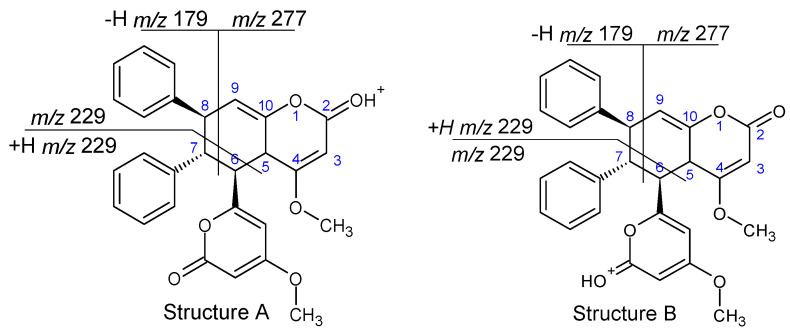

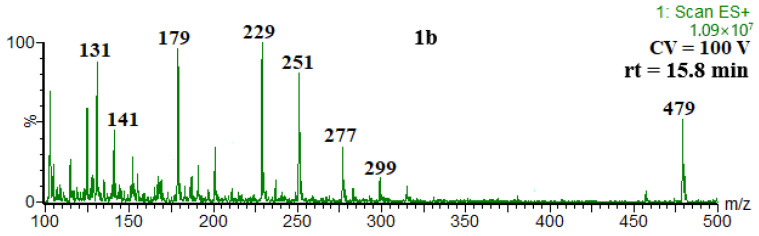
Plausible structures of protonated **1b**, main fragmentation pathways, and CID in-source mass spectrum.

More difficult was the elucidation of the structure of **1b**, its plausible structure (obviously, other geometric isomers are also possible) and its ESI mass spectrum obtained at a high cone voltage, which are shown in Figure 9. This compound has a completely different fragmentation pattern than **1a** and **1c** (although **1a** and **1b** yield product ions characteristic of **1**, Figure S7 and Figure 9); therefore, it cannot be a geometric isomer of any of them, and its structure must allow generation of the product ions at *m*/*z* 277, 229 and 179. The structure formed as a result of [4+2] cycloaddition, the well-known Diels–Alder reaction, meets these criteria. In order to further confirm the assumed structure of dimer **1b**, the quantum theoretical calculations of its protonated **1b** version were performed and compared to its observed fragmentation pathways. It is reasonable to assume that the most probable protonation sites of **1b** are carbonyl oxygen atoms, as confirmed by the quantum chemical calculation performed for monomer **1** (Supplementary Material, Table S1, Figure S9). The protonation of both carbonyl oxygen atoms of **1b** was found to be almost equally probable (Supplementary Material, Table S2, Figure S10). It was concluded that the observed fragmentation pattern of protonated **1b** consists in the rupture of the weakest bond of the bicyclic system (Table 1, Figure 9), which confirms the assumed structure of **1b**.

To the best of our knowledge, the reported earlier kavalactone dimers have been formed through [2+2] cycloaddition; therefore, they contained a four-membered ring, similarly to compounds **1a** and **1c** [5,35,36,37,38,39]. The tentatively identified product **1b** of [4+2] cycloaddition (Figure 9), to the best of our knowledge, is a new compound whose structure requires further confirmation.

#### 2.5.2. Confirmation of the Aglycone Structure of 4′-Hydroxy-5,6-dehydrokavain-4′-O-glucoside

**Figure 10:**
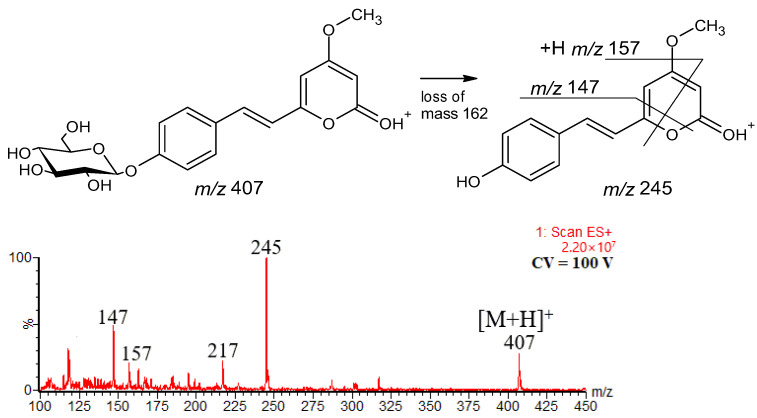
Fragmentation pathway and ESI mass spectrum of 4′-hydroxy-5,6-dehydrokavain-4′-O-glucoside.

The plant best known for the kavalactones occurrence is *Piper methysticum* (Kava) and *Alpinia zerumbet*, but these compounds also occur in a number of other plants, such as *Alpinia speciosa* [40], *Aniba species* [41,42], *Polygala species* [43,44] and *Achyrocline satureioides* [45]. In the extract of the latter (a herbaceous plant that exhibits various pharmacological properties), recently, the kavalactone glucoside has been identified on the basis of the [M+H]^+^ ion at *m*/*z* 407, and [aglycone+H]^+^ ion at *m*/*z* 245 (data obtained in the negative-ion mode also confirmed the presence of this compound) [21]. We found that subsequent decomposition of [aglycone+H]^+^ ion confirms the aglycone structure, as shown in Figure 10. The product ions at *m*/*z* 147 and 157 were formed in ways analogous to those of the formation of ions at *m*/*z* 131 and 141, which were observed for compound **1** (Figure 2 and Figure 3). In other words, the fragmentation pathways found for **1** also occur for its 4′-hydroxy conjugate, and even for the glycoside of this conjugate, allowing for the determination of the aglycone structure.

## 3. Materials and Methods

### 3.1. Preparation of the Analyzed Samples

Commercially available yangonin (**2**; Chemat, Gdańsk, Poland), (±)-kavain (**4**; AmBeed, Buffalo Grove, IL, USA) and 5,6-dehydromethysticin (**3**; LGC, London, UK) were used as standards and for syntheses.

Compounds **1**, **2**, **4**, **5**, **6**, **10**, **11** and **12** were identified in the extract of *Piper methysticum*. Extracts were prepared from commercially available dried and powdered Kava Kava rhizomes (Borongoru and Loa Waka varieties; Nanga brand, Blękwit, Poland). Briefly, 250 mg of the plant material was mixed with methanol (2.5 mL) and sonicated for 10 min at room temperature. The extracts were then filtered through a syringe filter (0.45 μm, PTFE membrane) and stored in a refrigerator prior to analysis.

Compound **7** was identified in the extract of *Alpinia zerumbet*. The extract was prepared from fresh leaves of *Alpinia zerumbet* cv. Variegata. The plant material was cut into pieces, frozen with liquid nitrogen, and ground in a mortar. Approximately 1 g of the ground leaves was mixed with 2.5 mL of methanol and sonicated for 10 min at room temperature. The extract was then filtered through a syringe filter (0.45 μm, PTFE membrane) and stored in a refrigerator prior to analysis.

Compounds **8** and **9** were prepared by the catalytic hydrogenation of yangonin or 5,6-dehydromethysticin, respectively, according to the procedure described by Soldi et al. [46].

### 3.2. HPLC-MS/MS Analysis of the Plant Extracts

The extracts of *Piper methysticum* and *Alpinia zerumbet* were analyzed by HPLC-MS/MS on an UltiMate^TM^ 3000 UHPLC system (ThermoScientific/Dionex, Sunnyvale, CA, USA) coupled with an Impact HD mass spectrometer (Bruker Daltonics, Billerica, MA, USA). The MS/MS spectra (collision energies 15, 20, 25 eV) were recorded in the *m*/*z* range 50–400 in the positive ion mode. The ESI source parameters were as follows: capillary voltage 3.6 kV, drying gas flow 8 L/min, nebulizer pressure 1.5 bar and drying gas temperature at 200 °C. The chromatographic separation was carried out on a Kinetex C18 column (2.6 μm, 100 mm × 2.1 mm i.d.; Phenomenex), which was maintained at 35 °C during the analysis. The mobile phase consisted of water with 0.1% formic acid (solvent A) and methanol with 0.1% formic acid (solvent B). The mobile phase was eluted in gradient elution mode, and the gradient was as follows: 0 min, 30% B; 1 min, 30% B; 20 min, 60% B; 21 min, 95% B; 23 min, 95% B; 24 min 30% B and 29 min, 30% B. The run time was 29 min and the sample injection volume was 1 μL. The mobile phase was pumped at a flow rate of 0.4 mL/min. The single-ion chromatograms obtained upon HPLC-MS/MS analysis are shown in the Supplementary Material (Figures S1 and S2). Although most of the kavalactones detected in the extract of *Piper methysticum* have not been well separated, the use of a high-resolution q-tof instrument allowed for the recording of the pure MS/MS spectrum (product ion spectrum) for each compound, which was not affected by the others. The so-called window width for the selection of precursor ion was 2 units; therefore, even for compounds **6** and **12** (*m*/*z* 275 and 277, respectively, Figure S1), it was possible to obtain the pure MS/MS spectra. Furthermore, the peak shapes are almost ideal, indicating no co-eluting isomeric or isobaric species (Figures S1 and S2). It has also been carefully checked whether, in the obtained MS/MS spectra, the isotope patterns of parent ions (the accurate *m*/*z* values and relative intensities of [M+H]^+^/[M+H+1]^+^) are in agreement with the calculated ones. It clearly confirmed the precursor ion selection and the lack of co-fragmentation.

### 3.3. Direct Infusion Analysis of Compounds ***3***, ***8*** and ***9***

The MS/MS spectra of compounds **3**, **8** and **9** were obtained using direct infusion. The sample solutions (methanol) were infused into the ESI source by a syringe pump at the flow rate of 3 µL/min. The instrument was operated under the following settings: end-plate voltage 500 V; capillary voltage 3.6 kV; nebulizer pressure 0.3 bar; dry gas (nitrogen) temperature 200 °C; dry gas flow rate 4 L/min; collision energies: 25 eV for sample **3** and 15 eV for samples **8** and **9**.

### 3.4. HPLC-MS Analysis of the Plant Extracts

Extracts of *Alpinia zerumbet* and *Achyrocline satureioides* (the latter was prepared according to the procedure described elsewhere [21]) were also analyzed by HPLC-MS cone voltage-induced fragmentation in-source (collision-induced dissociation in-source, CID in-source). The analyses were performed using a Waters Arc HPLC pump and a Waters SQD mass spectrometer (single-quadrupole-type instrument equipped with electrospray ionization (ESI) source, Z-spray, Milford, MA, USA). The software used was MassLynx V4.2 SCN1046 (Milford, MA, USA). Using an autosampler, the sample solutions were injected into the Atlantis C18 T3 column (3 μm, 100 mm × 3 mm i.d.). The injection volume was 5 µL. The solutions were analyzed using a linear gradient of CH_3_CN-H_2_O with a flow rate of 0.5 mL/min. The gradient started from 0% CH_3_CN to 95% H_2_O with 5% of a 10% solution of formic acid in water, reaching 95% CH_3_CN after 15 min, and the latter concentration was maintained for 10 min. The ESI mass spectra were recorded in the *m*/*z* range 70–700, in the positive-ion mode. The ESI source potentials were as follows: capillary, 3 kV; lens, 0.5 V; extractor, 4 V; cone voltage, 30–100 V. The proper choice of this parameter value has the greatest impact on the full-scan mass spectra recorded. An increase in this parameter leads to the so-called “in-source” fragmentation/dissociation, but a too-low cone voltage may cause a decrease in sensitivity. The source temperature was 120 °C, and the desolvation temperature was 300 °C. Nitrogen was used as the nebulizing and desolvating gas at flow rates of 100 and 300 L h^−1^, respectively.

### 3.5. Quantum Chemical Calculations

Full geometry optimizations (Supplementary Material, Tables S3 and S4) and energy calculations were performed within the DFT framework, at the B3LYP/6-311++G(d,p) [47,48] level of theory, because B3LYP (Becke, three-parameter, Lee–Yang–Parr) is one of the most popular functionals and can be applied for many different systems [49,50]. The 6-311++G(d,p) basis set (augmented with diffuse and polarization functions) is recommended for calculations performed for simple molecules that include electronegative elements and for comparisons with experiments [51]. All quantum chemical calculations were performed with GAUSSIAN 16 [52].

## 4. Conclusions

The fragmentation patterns of kavalactone [M+H]^+^ ions are sometimes very complicated, and for many product ions, it is difficult to propose their product ion structures. On the other hand, there are fragmentation pathways which are characteristic of given structures, and allow their identification including isomer differentiation. It has been clearly demonstrated that the characteristic product ions can be determined if the bonds between C5 and C6 atoms, and C7 and C8 atoms, are single or double. For example, benzylium ions are characteristic of compounds containing a single C7–C8 bond, indene—or indane-type ions—of compounds containing single C5–C6 bonds, R-C_6_H_5_-CH=CH-CO^+^ ions—of compounds containing double C5=C6 and C7=C8 bonds. The characteristic fragmentation pathways established for kavalactones have also been observed for kavalactone dimers (with the exception of dimer **1c**, which does not show the fragmentation pathways found for kavalactone monomers) and for kavalactone glycoside, irrespective of the instrumental conditions (CID in-source or CID-MS/MS). In other words, the fragmentation pathways determined for analyzed kavalactones can be regarded as characteristic of the whole class of these natural compounds, and can be very useful during their untargeted analysis in various plant materials, such as in plants in which kavalactones have not yet been detected, or in the detection of adulterated kava marketed products [53].

## Figures and Tables

**Table 1:** Calculated bond orders in bicyclic system of protonated structures (A and B) of **1b**.

Bond	Bond Order (A)	Bond Order (B)
O1–C2	0.8569	0.7291
C2–C3	0.9110	0.7749
C3–C4	1.0456	1.1652
C4–C5	0.8419	0.8401
C5–C6	0.5799	0.6217
C6–C7	0.0742	0.1065
C7–C8	0.6677	0.6691
C8–C9	0.8421	0.8496
C9–C10	1.3696	1.3536
C10–O1	0.6114	0.7046

## Data Availability

The data presented in this study are available in this article.

## Supplementary Materials

The following supporting information can be downloaded at https://www.mdpi.com/article/10.3390/ijms27062840/s1.
