# Supplementary material for: Signals of Diagnostic Product Ions of Kavalactones in Their ESI Mass Spectra—Implications for Isomer Differentiation and Identification of Kavalactone Conjugates
Source: Int J Mol Sci. 2026 Mar 20;27(6):2840. doi: 10.3390/ijms27062840 (PMC13027372; doi:10.3390/ijms27062840)
Supplement: Supplementary file 1 [file ijms-27-02840-s001.zip › ijms-4173582-supplementary.pdf]

Signals of diagnostic product ions of kavalactones in their ESI mass spectra – implication for isomer differentiation and identification of kavalactone conjugates

SUPPLEMENTARY MATERIAL

Table of contents:

Figure S1. Single ion chromatograms of $[M+H]^+$ ions of kavalactones obtained for the extract of *Piper methysticum*. 2

Figure S2. Single ion chromatogram of $[7+H]^+$ ion obtained for the extract of *Alpinia zerumbet*. 2

Figure S3. MS/MS spectrum of *cis*-**2** isomer. 3

Scheme S1. Plausible fragmentation pathways of **1-3** corresponding to the loss of small molecules. 3

Scheme S2. Plausible fragmentation pathways of **4** and **5** corresponding to the loss of small molecules. 4

Scheme S3. Plausible fragmentation pathways of **10** corresponding to the loss of small molecules. 4

Figure S4. Single ion chromatogram of ion at *m/z* 457. 5

Figure S5. ESI mass spectrum of **1** obtained at low cone voltage. 5

Figure S6: ESI mass spectra of **1a**, **1b** and **1c** obtained at low cone voltage. 6

Figure S7. ESI mass spectrum of **1a** obtained at high cone voltage. 7

Figure S8. ESI mass spectrum of **1c** obtained at high cone voltage. 7

Table S1. Calculated energy and relative energy of protonated **1**. 8

Figure S9. The most energetically favoured optimized structure of protonated **1**. 8

Table S2. Calculated energies and relative energies of protonated structures of dimer **1b**. 9

Figure S10. The optimized structures of protonated **1b**. 9

Table S3. Atomic coordinates of protonated compound **1**. 10

Table S4. Atomic coordinates protonated dimer **1b**. 11

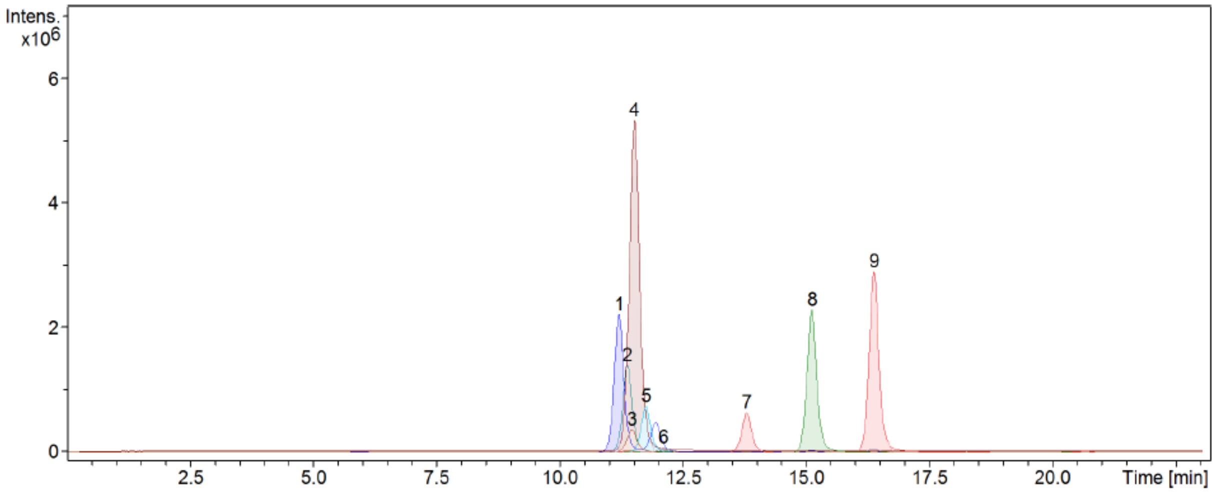

#	RT [min]	Area	I	Chromatogram	
1	11.2	30213790	2200306	EIC 231.1016±0.02 +All MS	[**4**+H]$^+$
2	11.4	19411516	1382319	EIC 277.1071±0.02 +All MS	[**12**+H]$^+$
3	11.5	5477141	361331	EIC 275.0914±0.02 +All MS	[**6**+H]$^+$
4	11.5	79873912	5307819	EIC 233.1172±0.02 +All MS	[**10**+H]$^+$
5	11.8	10314440	732470	EIC 263.1278±0.02 +All MS	[**11**+H]$^+$
6	12.1	1136140	66438	EIC 261.1121±0.02 +All MS	[**5**+H]$^+$
7	13.8	8945459	629673	EIC 259.0965±0.02 +All MS	[**2**+H]$^+$ *cis* isomer
8	15.1	31961696	2273015	EIC 229.0859±0.02 +All MS	[**1**+H]$^+$
9	16.4	40708988	2886319	EIC 259.0965±0.02 +All MS	[**2**+H]$^+$

Figure S1. Single ion chromatograms of [**M**+H]$^+$ ions of kavalactones obtained for the extract of *Piper methysticum*.

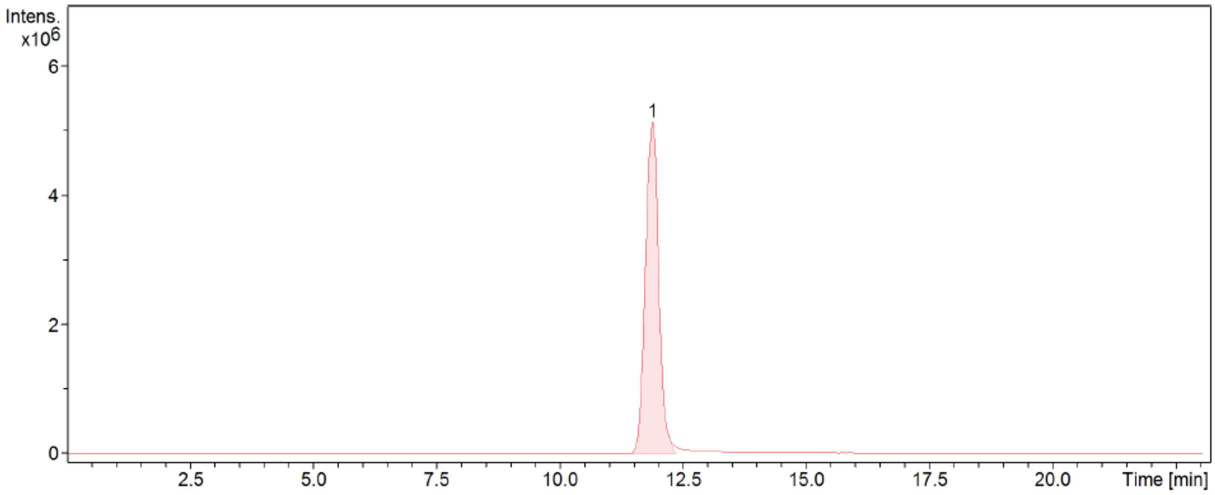

#	RT [min]	Area	I	Chromatogram	
1	11.9	96715456	5132810	EIC 231.1016±0.02 +All MS	[7+H]$^+$

Figure S2. Single ion chromatogram of [**7**+H]$^+$ ion obtained for the extract of *Alpinia zerumbet*.

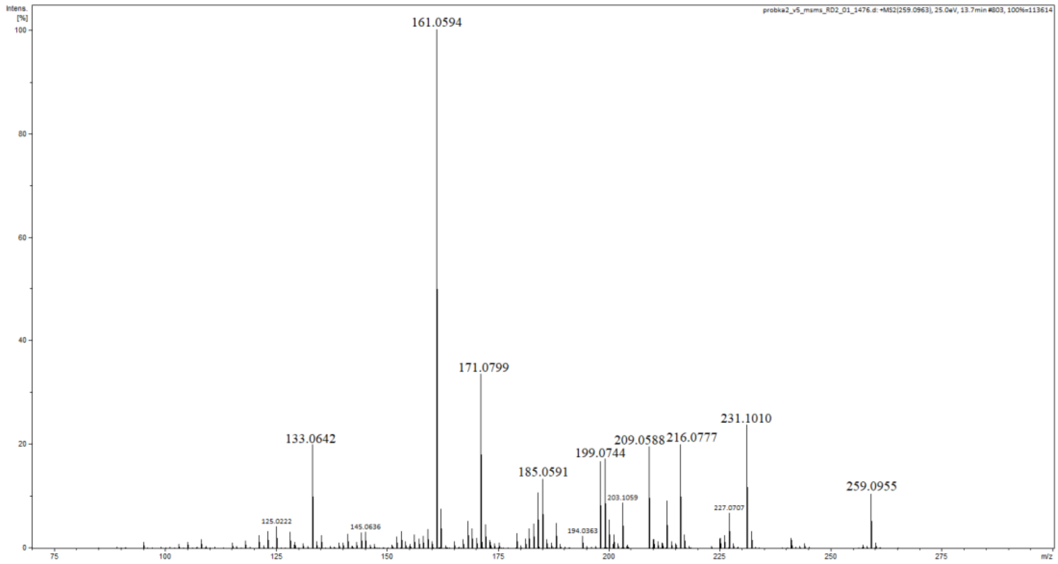

Figure S3. MS/MS spectrum of *cis*-**2** isomer.

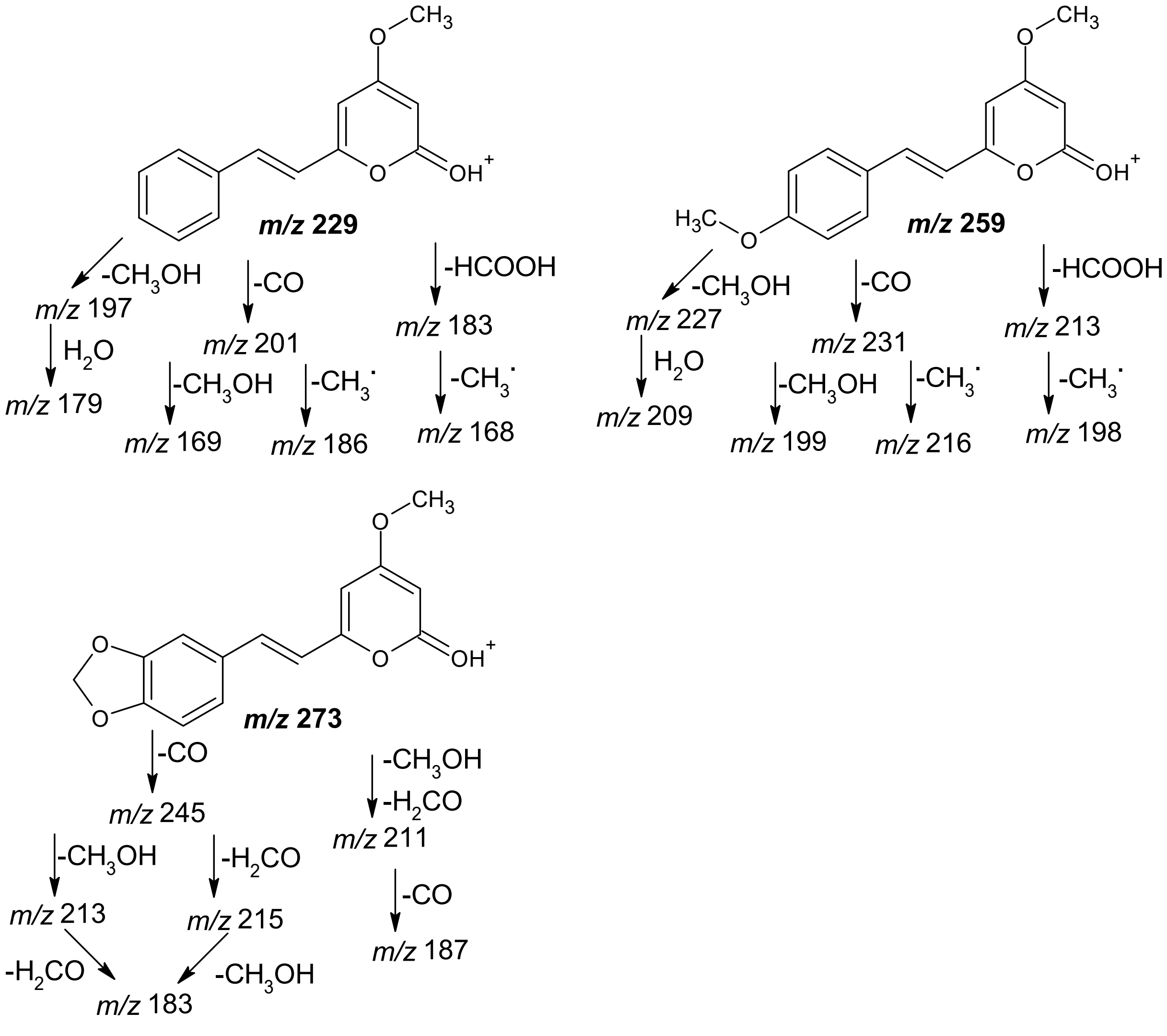

Scheme S1. Plausible fragmentation pathways of **1-3** corresponding to the loss of small molecules.

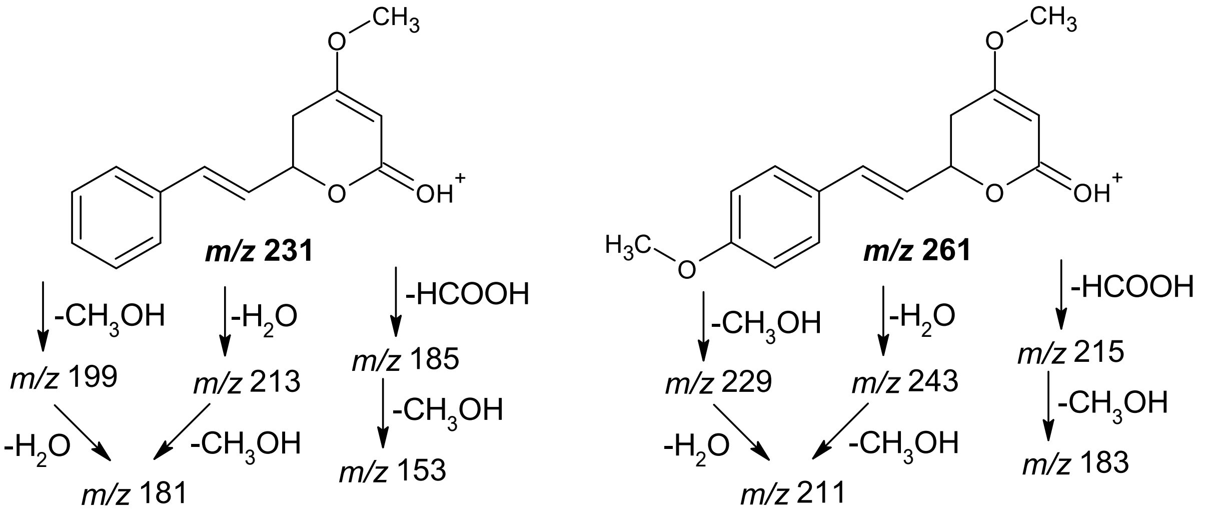

Scheme S2. Plausible fragmentation pathways of **4** and **5** corresponding to the loss of small molecules.

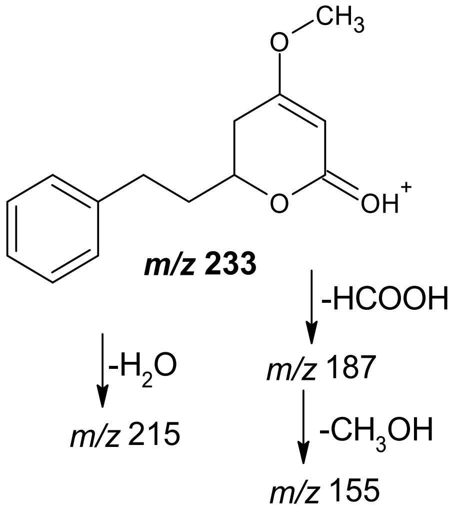

Scheme S3. Plausible fragmentation pathways of **10** corresponding to the loss of small molecules.

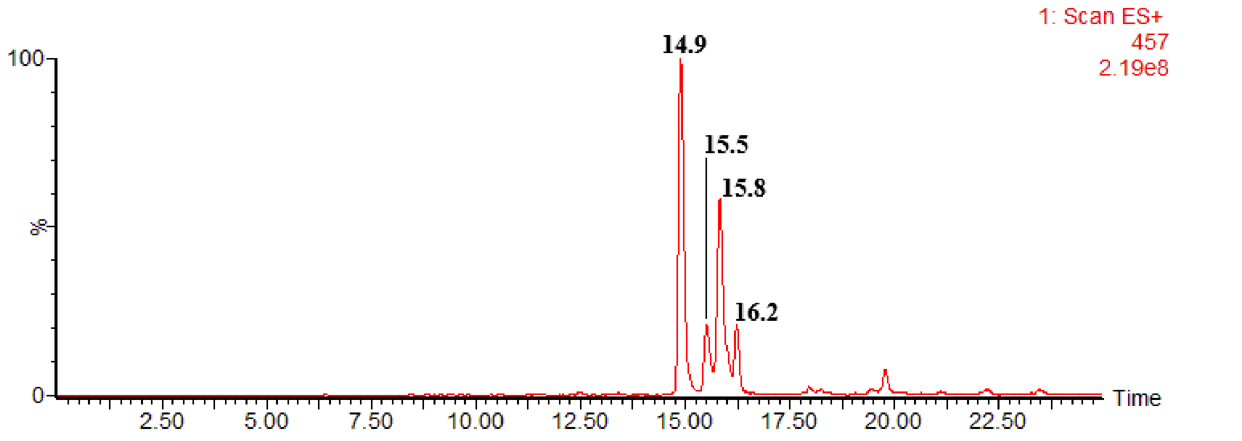

Figure S4. Single ion chromatogram of ion at *m/z* 457. Peaks at rt = 14.9 corresponds to the ion $[(\mathbf{1})_2\text{+H}]^+$, these at rt = 15.5, 15.8 and 16.2 to the ions $[\mathbf{1a}\text{+H}]^+$, $[\mathbf{1b}\text{+H}]^+$ and $[\mathbf{1c}\text{+H}]^+$, respectively.

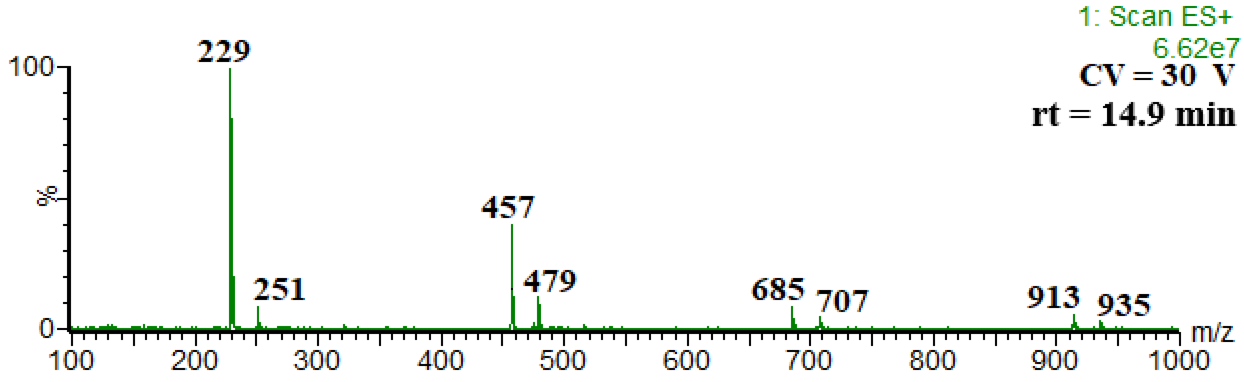

Figure S5. ESI mass spectrum of **1** obtained at low cone voltage. The observed peaks correspond the ions $[(\mathbf{1})_n\text{+H}]^+$ and $[(\mathbf{1})_n\text{+Na}]^+$ (n = 1-4).

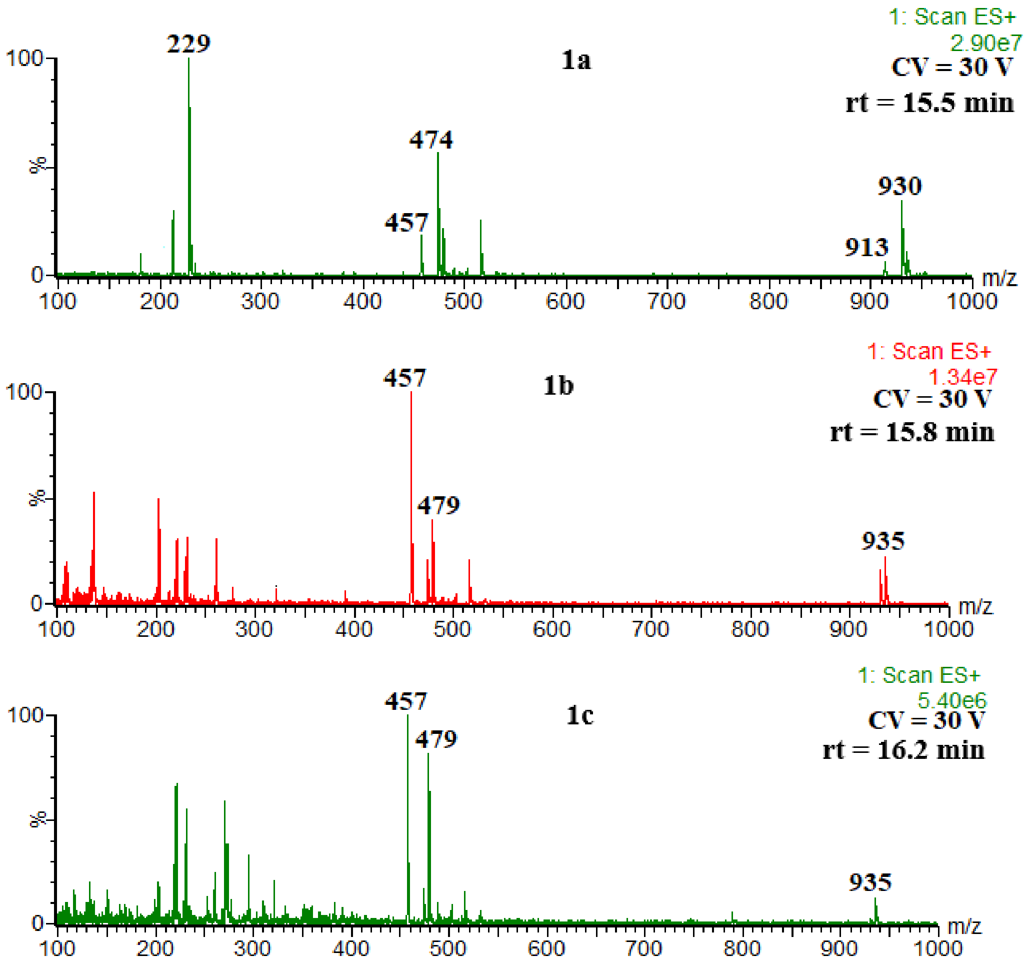

Figure S6. ESI mass spectra of **1a**, **1b** and **1c** obtained at low cone voltage. The adducts [**M**+H]$^+$, [**M**+NH_4]$^+$, [**M**+Na]$^+$ as well as [(**M**)$_2$+H]$^+$, [(**M**)$_2$+NH_4]$^+$ and [(**M**)$_2$+Na]$^+$ have been detected.

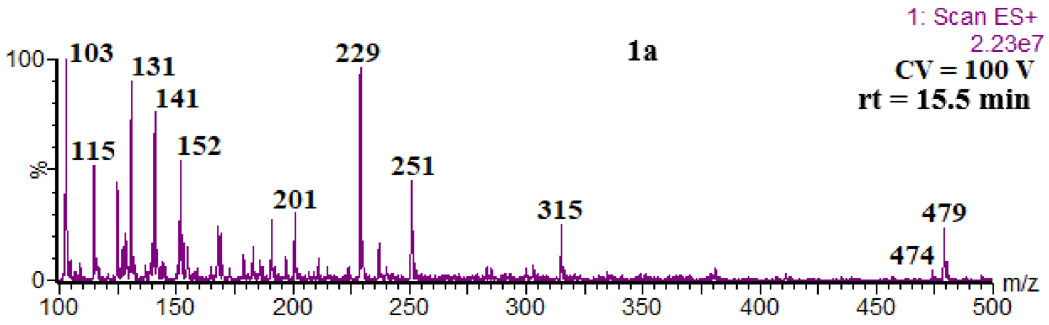

Figure S7. ESI mass spectrum of **1a** obtained at high cone voltage.

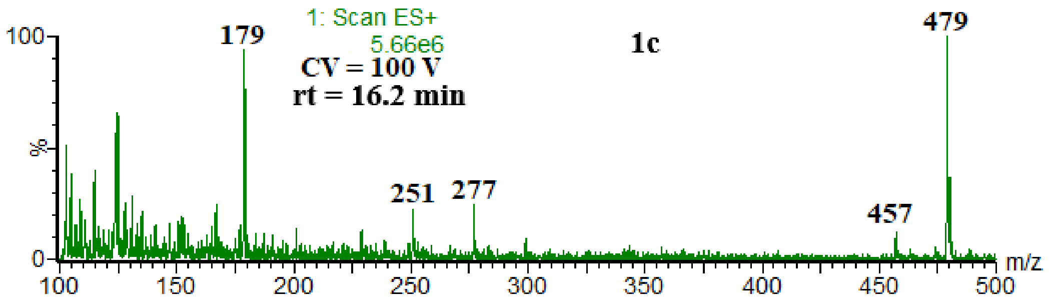

Figure S8. ESI mass spectrum of **1c** obtained at high cone voltage.

Table S1. Calculated energy and relative energy of protonated **1**.

Structure	Energy [Hartree]	Relative Energy [kcal/mol]
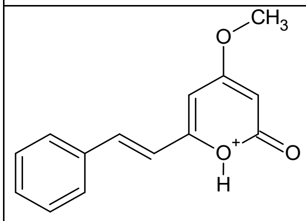	-766.905905	14.3
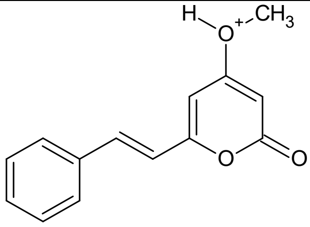	-766.858682	43.9
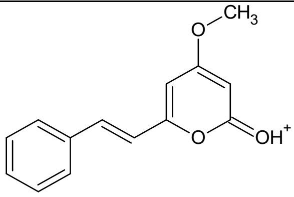	-766.928682	0.0[a]

[a]Absolute energy baseline [hartree]= -766.928682.

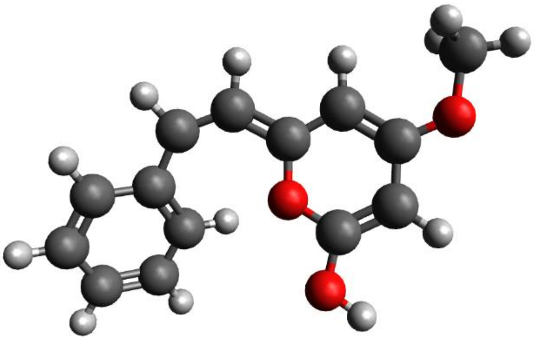

Figure S9. The most energetically favoured optimized structure of protonated **1**.

Table S2. Calculated energies and relative energies of protonated structures of dimer **1b**.

Structure	Energy [Hartree]	Relative Energy [kcal/mol]
A	-1533.509934	0.0
B	-1533.505033	3.1

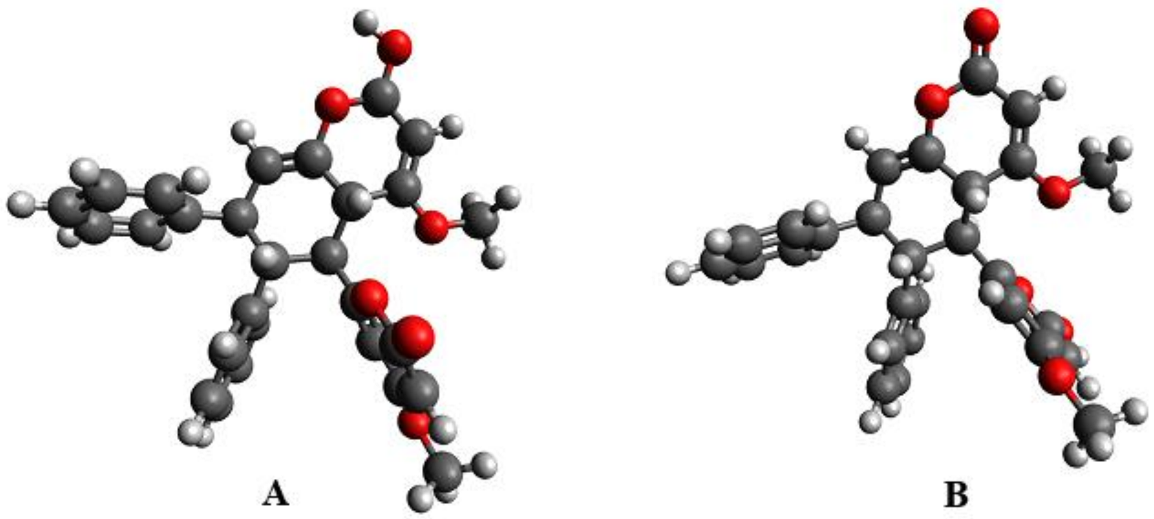

Figure S10. The optimized structures of protonated **1b**.

Table S3. Atomic coordinates of protonated compound **1**.

	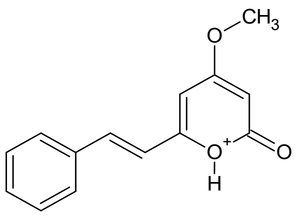			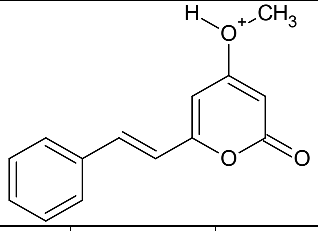			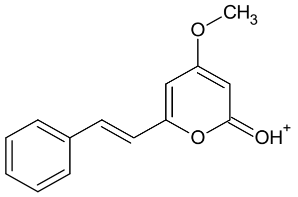		
**Atom**	**X**	**Y**	**Z**	**X**	**Y**	**Z**	**X**	**Y**	**Z**
C	4,955556	-45,0832	-2,16574	19,32823	-52,5642	-2,49072	30,02082	-52,0845	-2,43341
C	4,755272	-46,4741	-2,09013	18,52676	-53,5904	-1,93007	29,42752	-53,2702	-1,94629
C	4,418024	-44,3802	-3,2624	19,18431	-52,2963	-3,87123	29,77627	-51,7149	-3,77118
C	4,095577	-47,1475	-3,11074	17,63106	-54,3126	-2,70184	28,67816	-54,0879	-2,7796
C	3,740053	-45,06	-4,2727	18,2854	-53,0226	-4,63828	28,99832	-52,5179	-4,5915
C	3,590436	-46,4441	-4,20579	17,50774	-54,0296	-4,06257	28,46184	-53,7131	-4,1057
C	5,653009	-44,4018	-1,06744	20,2148	-51,9028	-1,55628	30,77119	-51,2911	-1,47862
C	6,628485	-43,4624	-1,11008	21,15729	-50,905	-1,55816	31,78579	-50,3795	-1,56769
C	7,409128	-43,0104	-2,24134	21,69833	-50,0392	-2,55398	32,6544	-50,0045	-2,63584
C	9,405828	-41,8508	-4,42683	22,70242	-48,3526	-4,59496	34,34896	-49,5198	-4,78867
C	8,795002	-42,7711	-5,19314	21,69859	-49,3402	-4,92697	33,34942	-50,4474	-4,8425
C	9,269791	-41,5349	-3,04797	23,1191	-48,3107	-3,30888	34,50678	-48,7891	-3,58855
O	7,261615	-43,5271	-3,46584	21,25292	-50,1306	-3,81647	32,53134	-50,6781	-3,81181
C	8,396472	-42,0641	-2,07148	22,68599	-49,1031	-2,24837	33,64699	-49,0471	-2,50915
O	10,05923	-40,6015	-2,53441	24,15624	-47,2963	-3,01399	35,47904	-47,9008	-3,58626
O	8,417376	-43,4766	-6,0076	21,20062	-49,5638	-5,98639	33,01805	-51,2306	-5,85049
C	11,05817	-39,8962	-3,30012	25,60127	-47,7048	-3,11339	35,74024	-47,0906	-2,41636
H	6,513792	-44,1528	-3,52583	23,95728	-46,8175	-2,18983	29,57289	-53,5493	-0,90837
H	5,13266	-47,0237	-1,23499	18,6185	-53,8139	-0,87278	30,15573	-50,7787	-4,15411
H	4,466458	-43,2964	-3,28572	19,76968	-51,5258	-4,34169	28,24768	-55,0042	-2,39446
H	3,962495	-48,2209	-3,04745	17,03055	-55,0919	-2,24855	28,79771	-52,2111	-5,61106
H	3,30924	-44,5052	-5,09773	18,1892	-52,8021	-5,69468	27,86024	-54,3389	-4,75449
H	3,062045	-46,9708	-4,99142	16,80863	-54,5903	-4,67223	30,45428	-51,4624	-0,45206
H	5,344564	-44,7092	-0,07077	20,10056	-52,3192	-0,55806	32,0609	-49,8937	-0,63801
H	6,955601	-43,042	-0,16642	21,60522	-50,7175	-0,58772	35,00315	-49,3376	-5,62983
H	10,13113	-41,3018	-5,01899	23,0475	-47,6882	-5,37329	33,73534	-48,5225	-1,57047
H	8,532082	-41,6517	-1,0817	23,0787	-49,026	-1,24489	33,59737	-51,0961	-6,61273
H	10,59018	-39,3179	-4,0993	25,71894	-48,0639	-4,13057	36,0171	-47,7244	-1,57231
H	11,52742	-39,2234	-2,58784	26,16982	-46,7954	-2,93779	36,57709	-46,4572	-2,69557
H	11,8004	-40,593	-3,69448	25,79087	-48,4786	-2,37337	34,86958	-46,4767	-2,1799

Table S4. Atomic coordinates protonated dimer **1b**.

	Structure A			Structure B		
**Atom**	**X**	**Y**	**Z**	**X**	**Y**	**Z**
C	25,40742	-38,3899	-1,84463	24,91374	-38,6246	-1,55996
C	25,6666	-39,8666	-1,7707	25,0908	-40,1237	-1,58802
C	24,29724	-37,7839	-2,2292	23,76974	-37,9859	-1,78215
C	24,57178	-40,6557	-2,55795	23,85889	-40,798	-2,24838
C	23,06592	-38,5401	-2,65151	22,44993	-38,6652	-2,04007
C	23,15276	-40,0472	-2,274	22,51776	-40,1844	-1,71082
C	21,81467	-37,8807	-2,07676	21,31493	-37,9953	-1,2695
C	22,04968	-40,8475	-2,95009	21,29606	-40,9416	-2,21034
C	20,78419	-37,4621	-2,92154	20,13371	-37,6309	-1,92063
C	19,63886	-36,8617	-2,40058	19,09102	-37,0269	-1,21918
C	21,68022	-37,6826	-0,69765	21,43471	-37,7373	0,101137
C	19,51259	-36,671	-1,02671	19,21751	-36,7805	0,14577
C	20,53704	-37,0826	-0,17518	20,39392	-37,1361	0,804794
C	21,95683	-40,9255	-4,34497	21,01198	-41,0501	-3,57801
C	21,09762	-41,5187	-2,17906	20,41364	-41,5317	-1,29956
C	20,07718	-42,2518	-2,78361	19,27895	-42,2136	-1,73868
C	19,99657	-42,3249	-4,17176	19,01039	-42,3176	-3,10155
C	20,94075	-41,6589	-4,95254	19,88105	-41,7328	-4,02086
C	27,70567	-37,9324	-1,58596	27,28188	-38,239	-1,70031
C	28,07368	-39,1914	-2,06746	27,39161	-39,5486	-2,35093
O	26,47749	-37,5401	-1,40824	26,03263	-37,8815	-1,22571
C	27,09327	-40,154	-2,19476	26,39609	-40,4479	-2,29098
C	24,58938	-42,1164	-2,17034	23,84386	-42,2816	-2,03548
C	24,51434	-43,1622	-3,01458	23,90069	-42,9479	-0,8527
C	24,46561	-44,4892	-2,46286	23,78533	-44,3584	-0,83169
C	24,51891	-44,6818	-1,10782	23,61383	-45,0507	-2,04286
O	24,63758	-42,2794	-0,83217	23,67802	-42,9819	-3,19142
C	24,62486	-43,573	-0,20408	23,56442	-44,3003	-3,19613
O	24,70945	-43,5748	0,992668	23,4131	-44,736	-4,4284
O	28,62843	-37,0595	-1,28302	28,1982	-37,4849	-1,53496
O	27,30727	-41,3726	-2,59628	26,43587	-41,7066	-2,77547
C	28,63873	-41,8265	-2,95996	27,63361	-42,1369	-3,44647
O	24,37307	-45,4569	-3,38342	23,84545	-44,9241	0,35305
C	24,28299	-46,8227	-2,9512	23,72545	-46,3588	0,500758
H	25,61065	-40,202	-0,72277	25,1863	-40,483	-0,55193
H	24,25585	-36,6998	-2,22042	23,77976	-36,9019	-1,75191
H	24,78117	-40,5834	-3,62849	23,91083	-40,6333	-3,32703
H	22,99957	-38,4562	-3,74274	22,21595	-38,5611	-3,10678
H	22,99954	-40,1198	-1,19327	22,54365	-40,2749	-0,61965
H	20,87084	-37,6119	-3,9925	20,02715	-37,8153	-2,9845
H	18,84633	-36,5454	-3,06877	18,18381	-36,7448	-1,74105

H	22,4693	-37,9967	-0,021	22,3545	-37,9869	0,621051
H	18,62257	-36,2046	-0,62099	18,41005	-36,3061	0,691214
H	20,44627	-36,9362	0,894969	20,50545	-36,9342	1,86405
H	22,67739	-40,4109	-4,97418	21,66695	-40,5913	-4,31187
H	21,14817	-41,4641	-1,09676	20,60006	-41,435	-0,23491
H	19,34417	-42,7606	-2,16825	18,59837	-42,6479	-1,01514
H	19,20256	-42,8919	-4,6435	18,12361	-42,8374	-3,44536
H	20,88283	-41,7072	-6,03401	19,67341	-41,7971	-5,08294
H	29,12185	-39,372	-2,24853	28,35276	-39,7521	-2,79903
H	24,45933	-43,0261	-4,0845	24,02	-42,4149	0,079562
H	24,49049	-45,657	-0,64647	23,51692	-46,125	-2,09453
H	28,23877	-36,2176	-0,99311	23,29781	-45,6952	-4,4645
H	28,50568	-42,8588	-3,26885	27,4445	-43,1592	-3,76568
H	29,02205	-41,2271	-3,78648	27,83062	-41,5071	-4,31663
H	29,29557	-41,7717	-2,09109	28,4849	-42,1108	-2,76264
H	25,18079	-47,1136	-2,40008	22,75293	-46,6948	0,136799
H	23,39593	-46,9717	-2,33098	24,54059	-46,8611	-0,02308
H	24,20106	-47,4107	-3,86178	23,80465	-46,5387	1,568789
